# The Impact of Prenatal Parental Locus of Control on Children's Psychological Outcomes in Infancy and Early Childhood: A Prospective 5 Year Study

**DOI:** 10.3389/fpsyg.2017.00546

**Published:** 2017-04-12

**Authors:** Stephen Nowicki, Yasmin Iles-Caven, Steven Gregory, Genette Ellis, Jean Golding

**Affiliations:** ^1^Department of Psychology, Emory UniversityAtlanta, GA, USA; ^2^Centre for Child and Adolescent Health, School of Social and Community Medicine, University of BristolBristol, UK

**Keywords:** ALSPAC, parental prenatal locus of control, child behavior, parenting skills, picky eating, sleep problems, temper tantrums

## Abstract

Locus of control is one of the most widely studied concepts in the history of personality psychology. In spite of its popularity and its associations with numerous relevant outcomes, the ability of locus of control to predict future behaviors involving parenting effectiveness has been under researched. The few parent locus of control children's outcome studies are characterized by cross-sectional methodologies that focus on mothers. The present study uses a prospective methodology to compare data on mothers' and fathers' locus of control with their child's behavior outcomes from a large scale research project, the Avon Longitudinal Study of Parents and Children (ALSPAC). Based on Rotter's Social Learning Theory published in 1954 and past empirical research, it was predicted and found that parent internality was associated with more positive child outcomes than parent externality. More specifically, when both parents were internal, their children had more positive outcomes in sleeping, eating, and tantrum behavior as compared to any other parent locus of control combination. However external parents had a less restrictive attitude which appeared to have a more beneficial effect on picky eating. Results confirmed how important parent locus of control is in the lives of children. Based on the findings, researchers are urged to develop interventions to change advice to parents and promote more internal locus of control among parents.

## Introduction

Fifty years ago Rotter (1966) published a study that has been cited in thousands of publications. In it, he introduced the concept of locus of control and provided a scale to measure it. He defined locus of control as a generalized problem solving expectancy.

“Internal vs. external control refers to the degree to which persons expect that a reinforcement or an outcome of their behavior is contingent on their own behavior or personal characteristics vs. the degree to which persons expect that the reinforcement or outcome is a function of chance, luck, or fate, is under the control of powerful others, or is simply unpredictable. Such expectancies may generalize along a gradient based on the degree of semantic similarity of the situational cues.” (Rotter, 1966).

His article has stimulated a remarkable amount of research over the past half century. A search of PsychInfo resulted in 17,812 articles with a keyword “locus of control” as of summer 2015, with 6,600 of these appearing after 1996 (1,425 between 2010 and 2015). Locus of control has sustained itself as a concept for psychological study for a half century (Nowicki and Duke, 2016).

During the half century since Rotter published his article others have offered a variety of definitions and methods for measuring the locus of control construct. Unfortunately some researchers have defined locus of control and used it in ways that are not consistent with Rotter's original definition and intent. Skinner (1996) found over 100 different definitions of locus of control and others (e.g., Furnham and Steele, 1993) have shown that scores from different locus of control scales often fail to correlate strongly or not at all with one another.

The tendency of researchers to be “creative” both in defining and developing measures of locus of control has made the task of generalizing findings across studies more difficult (see Furnham and Steele, 1993; Skinner, 1996). Some studies have used locus of control scales with so little evidence of construct validity that it is unclear what they are actually measuring. It is a tribute to the strength and viability of the locus of control construct itself that, in spite of the differences in definition and inconsistencies in measurement, findings have been replicated across an impressive collection of psychological and physical outcomes.

A complete review of locus of control findings is beyond the scope of the present paper. Suffice it to say that locus of control has been found to be associated with achievement, be it in *academia* (Kalechstein and Nowicki, 1997; Flouri, 2006), *sports* (Morris et al., 1979; Porat et al., 1989; Arnaud and Palazzolo, 2012), or *business* (Spector et al., 2002; Wu et al., 2015) as well as with indicators of *physical health* (Sørlie and Sexton, 2003; Sturmer et al., 2006; Chipperfield et al., 2016) and *psychological adjustment* (Harrow et al., 2009; Cheng et al., 2013). (For other reviews of locus of control findings see Lefcourt, 1982, 1983; Kormanik and Rocco, 2009; Nowicki, 2016a).

### Parent locus of control

In spite of its obvious importance, few of the many thousands of locus of control studies have directly investigated the association between parent locus of control and child outcomes. Unfortunately, the studies that have been completed are characterized by the application of cross-sectional methodologies, small numbers of participants, an emphasis on mothers' not fathers' locus of control, and an unfortunate propensity to measure locus of control in a variety of ways.

The most frequently used measure in this area of work is the Parent Locus of Control scale (Campis et al., 1986) but most often researchers pick out different subscales as their measure (e.g., total score, parent control, or parent efficacy scales). Two studies (Ollendick, 1979; Hoza et al., 2000) chose global locus of control scales and another provided a “new” test of locus of control but did not provide evidence of convergent or discriminative validity or reliability (e.g., Becker et al., 2010).

Another shortcoming in the research conducted regarding the association of parent locus of control and child outcomes is that researchers studied samples of children that varied widely in age. For example, Becker et al. (2010) included children between 6 and 14; Campis et al. (1986) used “elementary” school age children; Janssens (1994) studied children between 9 and 12 and McElroy and Rodriguez (2008) used children between 5 and 12. Preschool children constituted the most homogeneous age population studied (e.g., Estroff et al., 1994; Coyne and Thompson, 2011).

In spite of the limitations of these studies one consistent result was found; externality in one or both parents was associated with more negative outcomes in their children, whether the outcomes were measured in preschool (e.g., Estroff et al., 1994), or preadolescent and adolescent participants (e.g., Freed and Tompson, 2011), or by diagnoses like Attention Deficit/Hyperactivity (Hoza et al., 2000), or anxiety (Becker et al., 2010).

Three studies deserve additional mention. Two because they examined the impact of parent locus of control on children's behavior over time and one because it assessed both parents' global locus of control and used a system of combining their scores that made it easier to evaluate the contribution of each parents' orientation to their children's outcomes.

First, Moreland et al. (2016) examined outcomes in preschool children as a function of parent locus of control (as measured by the specific parental control subscale of the overall Parental Locus of Control scale) in both mothers and fathers. The authors were interested in determining if a parenting intervention would have a beneficial impact on both the stress level and locus of control of parents along with concomitant positive change in their children's behavior. Before the intervention, externality in mothers, and fathers was related to greater disruptive behavior and lower cognitive coping in children. However, the successful intervention lowered parental stress and made them more internal, children's disruptive behavior decreased and their coping skills increased. These findings suggest that interventions making parents more internal may also increase the likelihood that children's behavior will improve.

Second, while Hagekull et al. (2001) did not include an intervention, they did complete a long term prospective study on parent locus of control. They used two of the five subscales of the Parental Locus of Control scale (parental control and parent responsibility). Both subscales were completed when children were 33 months old and again when they were 9 years old. The researchers found that externality as measured on the parental control subscale for mothers and fathers was uniquely related to greater child difficulties both concurrently and prospectively. They concluded that “the results of the present study point to parents' perceived control as important for their children's development of externalizing and internalizing problems as well as for social and non-social competence development.”(p. 436). They went on to suggest that parent control perceptions had an independent impact on development during the preschool years over and above infant temperament and acting out behavior.

Although Ollendick (1979) did not include an intervention or use a prospective design, he did administer age appropriate forms of the same generalized locus of control scale to parents (Adult Nowicki Strickland Internal-External control scale, Nowicki and Duke, 1974) and children (Children's Nowicki Strickland Internal External control scale. Nowicki and Strickland, 1973). He then organized them into different combinations based on their locus of control scores: Mother and father both internal, mother internal-father external, father internal-mother external, and mother and father both external. Forming these combinations allowed him to evaluate the input of locus of control orientation for each parent on their child's behavior. He concluded that “children who have both external locus of control parents were significantly more anxious than other children who had at least one, or both internal locus of control parents. These results indicate the benefits of children having at least one internally controlled parent. It would seem most plausible that external locus of control parents are those who are the most inconsistent in their parenting attempts. The ill-effects of inconsistent parenting on children has been described by Lefcourt (1976).”

### The present study

Based on the definition of locus of control offered by Rotter (1954, 1966, 1975, 1990) and explicated by Lefcourt (1976, 1981, 1982, 1983), characteristics associated with internality should lead internals to be better parents than externals. Internals are theorized to be more responsible and persistent, more able to delay gratification and better able to gather relevant information than externals, all of which are advantageous for effective parenting. As mentioned earlier, research results confirm that, generally, internals achieve more, are better adjusted and more successful than externals.

Research on the parent locus of control, child outcome findings have supported theoretical assumptions; external parents have children who are more likely to have negative personality and behavioral outcomes than offspring of internal parents. However, previous findings were based on studies that failed to use prospective methodologies, representative populations of participants, reliable locus of control scales consistent with Rotter's definition and rarely included the locus of control of both parents.

The present study sought to remedy these shortcomings by using a prospective design with a large representative sample including both fathers and mothers, and a well-accepted Anglicized form of the Adult Nowicki Strickland scale to measure locus of control. In addition, because of the unique advantage of having both father's and mother's prenatal locus of control scores, four combinations of parent locus control could be formed identical to those used successfully by Ollendick (1979): Mother and father both internal, mother internal father external, mother external father internal, and mother and father both external. Based on social learning theory and past empirical research it was predicted that parents who were both internal would produce more positive child outcomes than parents who were both external.

More specifically the following predictions were made.

1. During pregnancy parents who are both internal compared to those who are both external would be more prone to seek out information about pregnancy and birthing and as a result be more likely to participate in “preparation” classes about giving birth.
2. Because parents who are both internal will be more organized, persistent and responsible than parents who are both external, they will be more prepared to solve the problems presented by their children's sleeping, eating, and tantrum behaviors and thus will have fewer difficulties in these areas.
3. In the parental pairing where one parent is internal and the other external, we suggest, consistent with what Ollendick found, that the presence of internality in at least one parent, especially the mother, will produce more positive outcomes when compared with both parents being external.
4. Because past research results have been mixed as to whether mothers' or fathers' locus of control differentially affect sons' and daughters' outcomes, we analyzed the data separately for boys and girls, without making predictions based on gender.

## Materials and methods

### Participants

The ALSPAC pre-birth cohort was designed to determine the environmental and genetic factors that are associated with health and development of the study offspring (Golding and the ALSPAC Study Team, 2004; Fraser et al., 2013). As part of the study design, therefore, there was a concerted effort before the child's birth to obtain from the parents details of their personalities, moods and attitudes, including a measure of their LOC.

ALSPAC recruited 14,541 pregnant women resident in Avon, UK with expected dates of delivery between 1st April 1991 and 31st December 1992 (an estimated 80% of the eligible population). Data were collected at various time-points using self-completion questionnaires, biological samples, hands-on measurements, and linkage to other data sets. The ALSPAC Ethics and Law Advisory Committee agreed that consent was implied if questionnaires were returned. Informed written consent was obtained for all biological samples prior to analysis, and for certain invasive procedures during the hands-on assessments (which were optional to attend).

With the advice of the ALSPAC Ethics and Law Advisory Committee it was decided not to enroll the study fathers directly, but rather to send to the mother a questionnaire for her partner and ask her if she would like her partner to be involved, and if so whether she would be good enough to pass the questionnaire to him (or her) with a separate reply-paid envelope for return. The study deliberately had no information on whether the mother had invited her partner to take part except when the completed questionnaire was returned. It should be noted that in consequence of this format, there was no way in which the study could send reminders to the partner themselves; during pregnancy questionnaires were returned by 76% of the partners of women who were taking part in the study.

For this project we have concentrated on the data collected from 9 questionnaires completed before and after the birth of the study child by the mother, and for the partner the questionnaire sent in pregnancy that included the LOC scale. The post-delivery questionnaires completed by the mother concerned the behavior of the child between 6 and 57 months post-delivery. The study website contains details of all the data that are available through a fully searchable data dictionary: http://www.bristol.ac.uk/alspac/researchers/data-access/data-dictionary/.

Ethical approval for the study was obtained from the ALSPAC Ethics and Law Committee and the Local Research Ethics Committees.

### Measures

#### Locus of control

The Adult Nowicki Strickland Internal External control scale (ANSIE, Nowicki and Duke, 1974) followed Rotter's definition in its construction. It has an easier reading level than the Rotter scale, but is significantly correlated with Rotter's test (Nowicki, 2016a) making it appropriate for testing adults from the general community.

An Anglicized and briefer form of the ANSIE was used in the present study. It contained the 12 items from the original 40 item scale which possessed the highest item-total correlations based on the responses of 135 mothers. Factor analysis of responses from 12,471 women confirmed the single factor structure of the scale. Coefficient alpha was 0.78 in this population. The scores ranged from 0 to 12 and were normally distributed with medians of 4 and 3 for the mothers (*n* = 12,471) and their partners (*n* = 8,645) respectively. The higher the score the more external the locus of control. For the present study external locus of control was defined as above the median while internal locus of control was defined as scores equal to or lower than the median.

#### Parental preparations and actions

There were six variables that we have used to describe the efficiencies of the parents during pregnancy and subsequently. Each, if carried out, has health benefits for the child. (i) During the pregnancy the mother was asked the date of her last menstrual period (LMP), and whether she was certain of this. From this the variable “uncertain of LMP” was derived to include those women who did not know their dates at all. After delivery the mother was asked (ii) whether she had attended labor and/or parentcraft classes during pregnancy; (iii) whether her partner had accompanied her; (iv) whether the father was present at delivery; (v) whether the child was breast fed; and (vi) whether the child had received the full set of early immunizations recommended.

#### Sleeping behavior

Sleeping behavior of the child was measured using a number of questions repeated in different questionnaires which the mothers completed at their leisure at home. The questions were phrased as follows: “In the past year has your child regularly (a) refused to go to bed; (b) got up after only a few hours' sleep?” In addition they were asked: “Does your child have a regular sleeping routine (i.e., does he/she tend to go to sleep at the same time every day)?” These questions were asked at ages 18, 30, 42, and 57 months of age.

#### Feeding behaviors

Feeding behaviors were elicited from the mothers with items used in the 1970 British birth cohort. They included the following questions in relation to the past year: (i) whether the mother found it difficult to establish an eating routine (asked at 24, 38, and 54 months), (ii) whether the child had over-eaten, and (iii) whether the child was choosy with food (15, 24, 38, and 54 months). At 57 months the mother was asked how much choice the child was allowed in regard to the main meal of the day, with three options for response: “from any available foods”; “from a few alternatives”; or “an adult decides.” She was also asked a similar question in regard to choice of snacks.

#### Temper tantrums

Temper tantrums were a focus of attention in questionnaires at 18, 30, and 42 months. When these occurred mothers were asked how often they reacted in particular ways (she ignored the child, tried to cuddle the child, reasoned with the child or tried to distract the child) with possible answers often; sometimes; never. When the child was 24 months old the mother was also asked how often she smacked the child, and/or shouted at the child when he/she was having a tantrum.

### Statistical approach

There is a considerable literature describing ways in which the child's adverse behavior is associated with maternal youth, low levels of education as well as exposure to prenatal smoking. These factors may also be a consequence of external LOC orientation—i.e., be on the causal pathway. Consequently analysis taking these three factors into account is likely to be misleading. We have therefore presented the data as unadjusted in order to demonstrate a pattern of associations.

For each child and parental outcome a comparison was made between maternal external and internal locus of control individuals, as well as between the paternal external and internal individuals. The comparisons of proportions with the outcome in each group used unadjusted chi-squared analyses and presented the results as odds ratios with 95% confidence intervals. These are all shown in the Supplementary Tables. For analyses assessing the differences within families, the proportion of each outcome is compared within the type of orientation of the mother. Thus, External mothers were selected and comparisons made between the outcomes according to the orientation of her partner; separately Internal mothers were selected and comparison of outcomes made between those whose partners were external and internal. For space reasons we did not present the odds ratios and confidence limits in these tables; the data are provided from which the interested reader can calculate these statistics if required.

The analyses were carried out for all children and, although we stratified by sex of the child, the results did not differ and have therefore been omitted from this paper.

## Results

### Parental preparations and actions

A comparison of externally and internally oriented mothers shows that those who were externally controlled were less likely to know the date of their LMP, less likely to attend parenting classes, less likely to breast feed and less likely to ensure their child was fully immunized by 6 months of age. Similar relationships for fathers with an external LOC were found to be associated with paternal failure to accompany the mother to classes or be present at delivery (Table S1).

An examination of the relationships when both parents were considered together (Table 1) showed that if the woman was externally oriented, the internal orientation of her partner appeared to have a beneficial effect on the mothers' knowledge of her LMP, whether the child was breast fed or fully immunized. If, however the mother was internally oriented, when compared with having an internal partner having an external partner appeared to result in more uncertainty about her LMP, being less likely to attend parenting classes, breast feed or ensure the child was fully immunized. The attendance of the father at classes, and (to a lesser extent) at delivery appears to have depended on the orientation of both parents, and was particularly likely if both were internal compared with both external.

**Table 1:** **The proportions (%) of parents who failed to undertake recommended health behaviors according to the LOC orientation of the pair**.

**Behavior**	***N***	**M.E+F.E %**	**M.E+F.I %**	***P***	***N***	**M.I+F.E %**	**M.I+F.I %**	***P***
Mother uncertain of her LMP	3,521	19.6	14.6	<0.001	4,984	20.5	9.6	<0.0001
Mother did not attend classes	3,288	42.7	39.1	0.044	4,680	37.1	31.7	<0.001
Father did not attend classes	3,275	77.4	71.1	<0.0001	4,660	70.4	60.4	<0.0001
Father not at delivery	3,302	14.7	12.2	0.038	4,688	9.8	7.3	0.002
Child not breast fed	3,407	34.9	24.3	<0.0001	4,773	20.1	10.1	<0.0001
6 months child not fully immunized	3195	16.9	11.8	<0.0001	4,646	10.0	7.4	0.002

*M.E, Mother external; M.I, Mother internal; F.E, Father external; F.I, Father internal; LMP, date of last menstrual period*.

### Sleeping

There are a variety of measures of the child's sleep behavior in the first 5 years of life, and we concentrate on the answers to three different questions answered by the mother at four different time points (18, 30, 42, and 57 months).

#### Children with no regular sleeping routine

The proportion of children without a regular sleeping routine gradually reduced between 18 and 57 months, but was always greater if the mother or the father was externally oriented (Table S2). In Table 2 we compare the responses of the four different combinations of parent locus of control: Both external (M.Ext+F.Ext), with mother external, father internal (M.Ext+F.Int), and mother internal, father external (M.Int+F.Ext), with both internal (M.Int+F.Int). It can be seen that: (i) if the mother is external, the proportion of children without a regular sleep pattern reduces if the father is internal; (ii) conversely if the mother is internal the proportion without a regular sleep pattern is greater if the father is external rather than internal; (iii) If both parents are external the risk of failure to have a regular sleep pattern is far greater than if both parents are internal—the ratio of the proportion of external to internal pairs is 1.88, 2.35, 2.73, and 2.93 for the ages 18, 30, 42, and 57 months respectively; (iv) for children who have one internal and one external parent the risk of failing to have a regular sleeping routine is greater for those for whom the mother is external, especially when aged over 18 months.

**Table 2:** **Percentage of parents who have sleeping problems with their child according to the locus of control orientation of both parents**.

**Age**	***N***	**M.E+ F.E %**	**M.E+F.I %**	***P***	***N***	**M.I+F.E %**	**M.I+F.I %**	***P***
**NO REGULAR ROUTINE**								
18 months	3,056	17.5	13.0	<0.001	4,536	13.5	9.3	<0.0001
30 months	2,855	16.0	12.4	0.007	4,282	10.0	6.8	<0.001
42 months	2,760	12.0	8.8	0.007	4,217	6.0	4.4	0.018
57 months	2,610	8.5	6.9	0.138	4,043	4.2	2.9	0.037
**REFUSAL TO GO TO BED**								
18 months	3,061	29.8	27.0	0.086	4,525	24.5	20.2	<0.001
30 months	2,846	55.4	47.5	<0.0001	4,280	44.0	36.1	<0.0001
42 months	2,783	51.8	44.3	<0.001	4,246	40.6	31.8	<0.0001
57 months	2,609	50.1	41.0	<0.0001	4,040	35.6	29.2	<0.0001
**GETS UP AFTER ONLY A FEW HOURS OF SLEEP**								
18 months	3,061	26.4	21.8	0.003	4,525	19.5	15.2	<0.001
30 months	2,824	27.6	25.3	0.188	4,261	21.2	16.1	<0.0001
42 months	2,783	21.8	19.1	0.087	4,246	16.4	11.6	<0.0001
57 months	2,602	15.5	12.8	0.050	4,024	12.2	8.1	<0.0001

*M.E, Mother external; M.I, Mother internal; F.E, Father external; F.I, Father internal*.

#### Refusal to go to bed

As with failure to have a regular sleeping routine, the proportion of children who refused to go to bed was higher if either parent had an external rather than an internal orientation (Table S3); this was true for both boys and girls. Comparison within the four combinations of parental locus of control (Table 2) shows a similar pattern to that found for a lack of a sleeping routine: (i) if the mother is external, the proportion of children refusing to go to bed reduces if the father is internal; (ii) conversely if the mother is internal the proportion refusing to go to bed is greater if the father is external rather than internal; (iii) If both parents are external the risk of failure to have a regular sleep pattern is far greater than if both parents are internal—the ratio of the proportion of external to internal pairs is 1.48, 1.53, 1.63, and 1.72 for the ages 18, 30, 42, and 57 months respectively; (iv) for children who have one internal and one external parent the risk of failing to have a regular sleeping routine is greater for those for whom the mother is external.

#### Gets up after only a few hours of sleep

In regard to getting up after a few hours' sleep the pattern is similar in that this is more likely to occur if the parent is external (Table S4). A study of the relationships with the LOC orientation of the two parents also shows a slightly different pattern to that found for the sleeping characteristics shown above. In Table 2 the rates of getting up after going to sleep indicate, once again, that if the mother is external, having an internal partner is associated with a reduced rate of this characteristic, but the relationships are significant in only one of the four ages. However if the mother is internal there is a more significant difference in this sleep pattern if her partner is external. Again the rate of getting up after only a few hours of sleep is much greater if both parents are external, compared to parents who are both internal, with ratios of 1.74, 1.71, 1.88, and 1.91; when there is a combination of externality/internality in the parents, the child will generally be slightly more likely to get up after a few hours of sleep if the father is internal.

### Feeding

Characteristics of the child's eating behavior were asked of the mother at 15, 24, 38, and 54 months post-delivery. Here we concentrate on three of these—the mothers' failure to establish an eating routine (unfortunately not asked at 15 months), the child perceived to have over-eaten and the child being choosy with food. The latter was chosen as it exhibits a reverse association with parental LOC orientation.

#### Mother finds it difficult to establish an eating routine

Difficulties with establishing a routine were greatest when the child was 38 months old, but at each age the difficulties were slightly greater if the parent was external (Table S5). Comparison of the four combinations of parents differing in locus of control, however, (Table 3) indicates that it is the mother's LOC that is important, and that of her partner appears to have no effect in this regard.

**Table 3:** **Percentage of parents who have feeding problems with their child according to the locus of control orientation of both parents**.

**Age**	***N***	**M.E.+F.E %**	**M.E.+F.I %**	***P***	***N***	**M.I.+F.E %**	**M.I.+F.I %**	***P***
**NO ESTABLISHED ROUTINE**								
24 months	2,887	23.0	23.1	0.956	4,365	20.8	20.6	0.864
38 months	2,803	28.2	26.0	0.182	4,272	24.3	23.5	0.585
54 months	2,662	23.9	22.0	0.257	4,049	18.0	17.4	0.650
**CHILD OVEREATS**								
15 months	3,075	22.5	20.2	0.133	4,521	21.1	16.9	<0.001
24 months	2,887	18.3	17.2	0.440	4,365	17.5	13.7	<0.001
38 months	2,803	15.8	14.0	0.197	4,272	15.3	12.0	0.003
54 months	2,668	21.1	17.2	0.013	4,054	16.3	14.1	0.054
**CHILD IS CHOOSY WITH FOOD**								
15 months	3,075	51.2	53.4	0.234	4,521	56.6	58.6	0.186
24 months	2,887	65.4	66.8	0.435	4,365	68.0	71.3	0.025
38 months	2,803	70.2	71.5	0.490	4,272	75.1	74.2	0.521
54 months	2,678	77.8	79.0	0.437	4,069	79.2	80.2	0.441

*M.E, Mother External; M.I, Mother Internal; F.E, Father External; F.I, Father Internal*.

#### Child overeats

The mother was asked on four occasions whether the child had had episodes of overeating in the past year. On each occasion the children with an external parent were more likely to be reported as over-eating (Table S6). In Table 3 we compare the four different combinations of parent locus of control. It can be seen that: (i) if the mother is external, the proportion of children who overeat reduces slightly but not significantly (except at 54 months) if the father is internal, (ii) conversely if the mother is internal the proportion of children that overeat is significantly greater if the father is external rather than internal, (iii) If both parents are external the risk of the child overeating is greater than if both parents are internal—the ratio of the proportion of external to internal pairs is 1.33, 1.34, 1.32, and 1.50 for the ages 15, 24, 38, and 54 months respectively, (iv) for children who have one internal and one external parent the risk of overeating is similar regardless of which parent is external.

#### Child has been choosy with food

Being choosy with food, also known as “picky eating” is common but can cause a great deal of stress to parents, and tends to develop into a habit that continues through childhood and beyond (e.g., Dovey et al., 2008; Taylor et al., 2015). At all ages the rate of this characteristic is lower if the mother is external although this pattern is much less apparent in relation to the father's orientation (Table S7). A comparison of rates of childhood choosiness within a family (Table 3) shows no consistent differences among external mothers if the father is internal, nor any difference among internal mothers if the father is external. Thus, the evidence suggests that it is only the mother's locus of control orientation and not the father's that is related to “choosiness,” and that unlike other types of feeding difficulties, it is the children of external mothers that have fewer problem behaviors.

In an attempt to investigate the reasons for these differences we compare the attitudes of the external and internal mothers and their partners in regard to allowing the child to choose what to eat. This indicates that the external mother is much more likely to allow a wide choice both of main meal and snacks compared to the mother with an internal LOC (Tables S8, S9). Comparison of the four different combinations of parent locus of control (Table 4) indicates that (i) if the mother is external she is less likely to allow choice of main meal from available foods if her partner is internal; (ii) she is also less likely to allow choice of all available snacks if her partner is internal; (iii) however if she is internally oriented the child is significantly more likely to be allowed to choose freely if the father is external; (iv) The child's ability to have a free choice of foods is greatest if both parents are external (19.7 and 51.4% for main meal and snacks respectively) and least if both are internal (8.8 and 30.2%); (v) if the partners are of mixed locus of control orientation, those with an external mother are more likely than those with an external father to allow free choice of main meal.

**Table 4:** **The choices presented to the child for the main meal and for snacks at 57 months according to the LOC orientation of the parents**.

**Choice**	**M.E+F.E %**	**M.E+F.I %**	***P* (*N*)**	**M.I+F.E %**	**M.I+F.I %**	***P* (*N*)**
**MAIN MEAL**						
From any available foods	19.7	16.6		11.4	8.8	
From a few alternatives	47.1	43.9		52.3	50.7	
Adult decides	33.3	39.5		36.3	40.6	
			0.001 (2562)			0.001 (3986)
**SNACKS**						
From any available snacks	51.4	42.4		39.6	30.2	
From a few alternatives	39.8	48.3		53.6	60.8	
Adult decides	8.8	9.3		6.8	9.1	
			<0.001 (2510)			<0.0001 (3924)

*M.E, Mother external; M.I, Mother internal; F.E, Father external; F.I, Father internal*.

### Temper tantrums

#### Prevalence of temper tantrums

The study mothers were asked whether their child had temper tantrums. Table 5 indicates that there is very little difference in the prevalence of tantrums between the children of mothers or fathers relative to their LOC orientation, although the proportion was always slightly higher for the children of externally orientated parents.

**Table 5:** **Proportion of children reported to have temper tantrums according to whether the parent was externally or internally oriented in pregnancy**.

**Age**	**N.Mother**	**M.E %**	**M.I %**	**N.FATHER**	**F.E %**	**F.I %**
18 months	10,571	89.2	87.5	7,590	89.4	86.4
30 months	9,892	89.2	88.8	7,163	88.8	88.4
42 months	9,610	84.0	81.8	6,994	83.6	81.7
57 months	9,146	76.6	72.4	6,690	74.9	72.6

*M.E, Mother external; M.I, Mother internal; F.E, Father external; F.I, Father internal*.

#### Actions of the mother when the child has a temper tantrum

Mothers were asked the same four questions at three time points (18, 30, and 42 months) concerning how they reacted when the child had a tantrum: (i) whether they ignored it; (ii) whether they cuddled the child; (iii) whether they tried to reason with the child; and (iv) whether they tried to distract the child. For each item they were given the options: Yes often; yes sometimes, and never.

#### Mothers ignore temper tantrum

The greater the presence of parent externality rather than internality the more likely it was that the mother would ignore the tantrum (Table S10). When the pair of parents were considered together there was little evidence that the study father had an influence on this behavior which was dominated by the mothers' externality (Table 6).

**Table 6:** **Frequency with which parents ignore temper tantrum**.

	**M.E.+F.E %**	**M.E.+F.I %**	***P* (*n*)**	**M.I.+F.E %**	**M.I.+F.I %**	***P* (*n*)**
**AT 18 MONTHS**						
Often	50.5	50.4		47.1	45.4	
Sometimes	44.2	42.9		44.8	45.9	
Never	5.3	6.7		8.0	8.7	
			0.499 (2711)			0.260 (3927)
**AT 30 MONTHS**						
Often	44.8	43.5		42.9	37.5	
Sometimes	51.6	51.6		51.5	56.2	
Never	3.6	4.8		5.6	6.3	
			0.271 (2487)			0.003 (3719)
**AT 42 MONTHS**						
Often	38.5	35.1		33.5	30.8	
Sometimes	52.9	54.8		56.9	59.1	
Never	8.6	10.1		9.6	10.1	
			0.060 (2325)			0.134 (3437)

*M.E, Mother External; M.I, Mother Internal; F.E, Father External; F.I, Father Internal*.

#### Mother cuddles the child during temper tantrum

There were consistent findings at each age such that externally oriented parents were more likely to state that they never cuddled the child when he/she was having a tantrum (Table S11). Results for the mother-father partnerships (Table 7) indicate that if the mother is external, having an internal partner makes a difference in that she is more likely to cuddle the child at least sometimes (74.0 vs. 69.6; 78.4 vs. 76.2; 70.1 vs. 65.6 for the three ages); if the mother is internal there is a similar but more marked contrast between her behavior when the fathers are external and internal (75.0 vs. 78.1; 78.6 vs. 81.8; 70.2 vs. 74.4).

**Table 7:** **Frequency with which child was cuddled during temper tantrum, comparing the orientation of each parent**.

**Frequency**	**M.E+F.E %**	**M.E+F.I %**	***P* (*n*)**	**M.I+F.E %**	**M.I+F.I %**	***P* (*n*)**
**AT 18 MONTHS**						
Often	15.1	16.8		16.5	19.8	
Sometimes	54.6	57.2		58.5	58.3	
Never	30.4	26.0		25.0	21.9	
			0.018 (2711)			0.003 (3927)
**AT 30 MONTHS**						
Often	16.5	17.4		16.3	18.0	
Sometimes	59.6	61.0		62.3	63.8	
Never	23.8	21.6		21.4	18.2	
			0.225 (2427)			0.020 (3639)
**AT 42 MONTHS**						
Often	12.1	12.3		12.3	13.5	
Sometimes	53.5	57.8		57.9	60.9	
Never	34.4	29.9		29.8	25.6	
			0.084 (2325)			0.016 (3437)

*M.E, Mother External; M.I, Mother Internal; F.E, Father External; F.I, Father Internal*.

#### Mother reasons with the child having a temper tantrum

In contrast with the other associations with behavior of the mother when the child is having a tantrum, there were no differences between the LOC orientation of either parent in regard to the mother trying to reason with the child (Table S12). Similarly there was no indication that the mother's behavior differed with the orientation of her partner (Table 8).

**Table 8:** **Frequency with which mother reasons with the child while having a temper tantrum**.

**Frequency**	**M.E+F.E %**	**M.E+F.I %**	***P* (*n*)**	**M.I+F.E %**	**M.I+F.I %**	***P* (*n*)**
**AT 18 MONTHS**						
Often	20.8	22.6		23.6	20.6	
Sometimes	56.1	51.5		54.0	54.8	
Never	23.1	25.9		22.4	24.5	
			0.710 (2711)			0.025 (3927)
**AT 30 MONTHS**						
Often	33.1	33.4		35.0	33.1	
Sometimes	59.0	59.7		58.1	59.3	
Never	7.9	6.9		7.0	7.6	
			0.590 (2441)			0.218 (3685)
**AT 42 MONTHS**						
Often	35.8	36.0		38.3	35.1	
Sometimes	53.8	55.9		53.2	56.4	
Never	10.4	8.1		8.5	8.6	
			0.334 (2325)			0.119 (3437)

*M.E, Mother External; M.I, Mother Internal; F.E, Father External; F.I, Father Internal*.

#### Mother tries to distract the child during temper tantrum

The frequency with which the mother tried to distract the child during a tantrum was strongly associated with the LOC orientation of each parent, with the children of internal parents being more likely to experience this often, and the children of external parents being at increased risk of never experiencing this (Table S13). This pattern was highly significant.

When the partnerships were analyzed (Table 9) it can be seen that the greatest rate of maternal distraction of the child was when both parents were internal compared with both being external (54.7 vs. 38.1% at 18 months; 42.9 vs. 33.6% at 30 months; and 31.5 vs. 25.3% at 42 months). Conversely the parents who are both external compared with those who are both internal have greater associations with the mother never trying to distract the child (14.1 vs. 5.6%; 10.4 vs. 4.3; 18.7 vs. 11.2%). Where the two partners are of different orientations there is some indication that the maternal orientation is more important, but this is not consistent at each age.

**Table 9:** **Frequency with which mother tries to distract the child during temper tantrum according to the orientation of each of the parents**.

**Frequency**	**M.E+F.E %**	**M.E+F.I %**	***P* (*n*)**	**M.I+F.E %**	**M.I+F.I %**	***P* (*n*)**
**AT 18 MONTHS**						
Often	38.1	44.6		50.5	54.7	
Sometimes	47.8	45.7		41.4	39.7	
Never	14.1	9.7		8.2	5.6	
			<0.0001 (2711)			<0.001 (3927)
**AT 30 MONTHS**						
Often	33.6	39.8		40.5	42.9	
Sometimes	56.0	52.5		51.8	52.8	
Never	10.4	7.7		7.7	4.3	
			<0.001 (2247)			0.004 (3715)
**AT 42 MONTHS**						
Often	25.3	26.1		30.1	31.5	
Sometimes	56.0	58.5		55.3	57.3	
Never	18.7	15.4		14.5	11.2	
			0.140 (2325)			0.034 (3437)

*M.E, Mother External; M.I, Mother Internal; F.E, Father External; F.I, Father Internal*.

#### Mother smacks and/or shouts at child

Only at one age (18 months) was the mother asked how often she smacked the child when he/she was having a tantrum. The mother was more likely to smack the child if she was external rather than internal. A similar pattern was shown for the orientation of the father (Table S14). Comparison of the mothers' actions depending on the orientation of the father indicates that there was no difference if the mother was external, but if she was internal, her partner's external orientation appears to have an influence such that she was more likely to slap the child (Table 10). The analyses for the mother shouting at the child were similar to those found for slapping (Table S15 and Table 11).

**Table 10:** **Frequency mother smacks the child when having a tantrum (18 months)**.

**Frequency**	**M.E+F.E %**	**M.E+F.I %**	***P* (*n*)**	**M.I+F.E %**	**M.I+F.I %**	***P* (*n*)**
Often	1.2	0.6		0.8	0.3	
Sometimes	25.8	26.3		22.4	18.4	
Never	73.0	73.1		76.8	81.2	
			0.709 (2711)			<0.001 (3927)

*M.E, Mother External; M.I, Mother Internal; F.E, Father External; F.I, Father Internal*.

**Table 11:** **Frequency mother shouts at the child when having a tantrum (18 months)**.

**Frequency**	**M.E+F.E %**	**M.E+F.I %**	***P* (*n*)**	**M.I+F.E %**	**M.I+F.I %**	***P* (*n*)**
Often	4.4	3.9		3.4	1.9	
Sometimes	59.2	60.3		53.8	50.8	
Never	36.4	35.8		42.7	47.2	
			0.984 (2711)			<0.001 (3927)

*M.E, Mother External; M.I, Mother Internal; F.E, Father External; F.I, Father Internal*.

#### Frequency of temper tantrums at 57 months

We have assessed the frequency with which children are having temper tantrums prior to reaching age 5, primarily to determine whether there were still differences between the children of external and internal parents. The children of mothers who were externally oriented were more likely to have temper tantrums, and to have them more frequently, than children of internally oriented women (Table 12). A similar pattern was shown with the orientation of the father. In general a comparison of the children of externally oriented women with external partners and internal partners showed only a small difference in frequency (*P* = 0.021); similarly among women who were internal, there was only a small difference associated with the father's orientation (*P* = 0.024).

**Table 12:** **Frequency with which child was having temper tantrums at 57 months**.

**Frequency**	**M.E+F.E %**	**M.E+F.I %**	**OR[95%CI]**	**M.I+F.E %**	**M.I+F.I %**	**OR[95%CI]**
1+ per day	3.1	1.9	1.76 [1.04, 2.99]	1.9	1.3	1.54 [0.91, 2.58]
Most days	15.5	12.1	1.40 [1.09, 1.79]	9.6	8.6	1.13 [0.90, 1.43]
1+ per week	22.1	23.0	1.05 [0.85, 1.29]	20.8	19.3	1.10 [0.92, 1.31]
<1 per week	35.5	38.8	1.00 Ref	41.1	42.0	1.00 Ref
Not at all	23.8	24.2	1.08 [0.88, 1.32]	26.7	28.7	0.95 [0.81, 1.11]
			*P* = 0.021 (*n* = 2616)			*P* = 0.024 (*n* = 4058)

*M.E, Mother External; M.I, Mother Internal; F.E, Father External; F.I, Father Internal*.

## Discussion

Being a parent is a most complex and demanding task. Children present a never ending progression of behavioral challenges for parents and how these demands are met determines their physical, psychological, and emotional development. The present study looked at some of the most important and complex parenting tasks to see how parents' locus of control appeared to influence children's responses. Developmental situations are complex and no single parental response will always be the best, but some responses are better than others for children over the long term. From what is known about locus of control, parental internality was expected to be associated with positive child outcomes more often than parental externality.

The results of this study reveal that internal and external parents approach the future birth of their child and his or her development in significantly different ways. From attitudes and beliefs expressed prenatally and on most indices of children's behavior and personality through infancy and toddlerhood, children of internal parents behaved differently from those of external parents. Overall the differences suggest internal as compared to external parents are more involved in acquiring relevant information about parenting skills and more organized and consistent in interactions with their children.

### Prenatal attitudes and behavior

Even before the baby is born, internal parents are more involved in finding out relevant parenting information than are external caretakers. For example, they were more likely to attend child preparation classes and their partners were more likely to accompany and to be present during labor and delivery than external parents-to-be. Internal as opposed to external mothers are more likely to know the date of their last menstrual period and to ensure their child is immunized by the age of 6 months. These behaviors are consistent with what is theoretically expected from internals (Rotter, 1966); that is, they, as internal parents, are more likely to seek out more information about the birth experience and subsequent child care than their external peers.

The core set of prenatal expectations held by internal parents and the information they gathered beforehand suggest that they also are better prepared for the arrival of the infant than external caretakers. Such preparation also indicates that they also may be more adept at dealing with future challenges presented by their children's sleeping, eating, and tantrum behaviors than external parents.

One early indication of this comes from the finding that internal as contrasted with external parents report being more willing to give their 4 week old babies “a cuddle” when they wake up at night. The act is consistent with the prenatal attitude of internal parents in which they stated they were more likely to adapt their lives to accommodate their new born than externals (data not shown).

### Sleeping behavior

Although we do not know all of the possible reasons for it, bed times are more problematic situations for children of external compared to internal parents. External parents appear less able to organize their children's lives and be consistent about procedures surrounding bedtime than internal caretakers. While we do not know what contributes to this outcome, we do know from past research with adults, that externals are less persistent and structured than internals (Nowicki, 2016b). If that is also true of their parenting behavior, then external parents may be less able to behave and parent consistently which, in turn, is more likely to create a confusing and unpredictable environment for their children, thus increasing chances for them to behave more negatively at bedtime.

### Feeding

A similar pattern of behavior surrounded the family's eating behavior as it had with sleeping situations. Externally controlled parents, especially externally controlled mothers, report a more difficult time establishing a family eating routine than their internal counterparts. Unsettled meal times may add to or perhaps cause the unsettled behaviors that seemed to surround and characterize bedtime for children of external mothers and fathers. With this in mind, it is not surprising that within this mealtime turmoil, children of external parents may eat more than they should. External parents compared to internal ones, are less likely to see the connection between their children's eating behavior and healthy outcomes and so they may also be less likely to monitor what and how much children are eating. Additional research that actually observes mealtime and bedtime situations is needed to establish exactly what transpires between parents and their children during these situations.

However, the data do reveal that children show markedly different patterns of association between the indicators of eating behavior and parent combination of locus of control. These can be summarized as follows: (i) difficulty in establishing an eating routine was governed primarily by the orientation of the mother, being higher if the mother was external; (ii) overeating was most prevalent if both parents were external; the greatest differences were found between them and both parents who were internal; (iii) if the mother was external the orientation of the father made little difference, however if the mother was internal the orientation of her partner significantly affected the eating behavior; (iv) children's choosiness about what to eat was most prevalent if the mother was internal; the orientation of her partner made little difference in this behavior; the evidence suggested that the externally oriented mothers had a laissez faire attitude to what their children ate, resulting in a lower prevalence of picky eating.

The fact that internal parents, especially mothers, reported their children as being “more choosy” about the food they ate deserves some comment. This could be a negative feature of being a child of internal parents in the sense that it reflects being “spoiled” or “catered too.” However, experts in the “picky eating” field show that insistence of parents in trying to guide their child to eat foods they do not like results in some adverse nutritional consequences and even increased prevalence of constipation (Taylor et al., 2016). Additional observational research focusing on exactly what and how the children of internal parents are offered food compared to children of external parents is necessary to resolve the possible explanations offered or to come up with new ones.

### Temper tantrums

The unpredictability and chaos that surrounds sleeping and eating routines is also present in how internal and external parents deal with their children's tantrums. Tantrums are a difficult problem for all parents, especially as children grow older. Often there is a public aspect to the tantrums that increases their impact; children's crying and screaming often draws the unwanted attention and scrutiny of others. From our data analysis it is apparent that regardless of locus of control orientation all parents have to face the stress of dealing with their children's tantrums and are equally prone to try to use “reasoning” to deal with them.

However internal and external parents differ in the use of other interventions to deal with their children's tantrums. For example, at 18 and 30 months of age, external as compared to internal parents are more likely to pay attention to occurrences rather than ignore them, and less likely to “hold/cuddle” their children and/or try to distract them as ways to deal with tantrums. External parents appear to be more likely to distance themselves from their children than their internal peers do and less prone to engage them by the use of soothing or distracting interventions. If this is what is occurring then external parents may be creating a more negative and perhaps even hostile interpersonal situation between them and their children during the tantrum behavior which may increase the intensity of the interaction. Support for this possibility is found in the responses of parents to two additional questions about their reaction to tantrums when their child was 18 months of age. External parents were significantly more likely to “shout at” or “smack/shake” their child during a tantrum than internal parents. The application of such extreme responses does not bode well for quietening the children or improving the parent child relationship. As with sleeping and eating behavior, it would be helpful to have observational data of the ongoing interactions between parents and children to evaluate the quality and the effectiveness of internal and external parents' interventions dealing with tantrums.

To summarize. Temper tantrums are common and are only slightly more prevalent if parents are external. However the behaviors of the mother when her child has a temper tantrum varies in regard to her locus of control and that of her partner in the following ways:

1. The mother ignores the temper tantrum—this behavior was more often found with external mothers. The fathers' locus of control seemed to make no impact.
2. Cuddling the child was less likely to be a strategy if either parent was external.
3. Reasoning with the child was not associated with the locus of control orientation of either parent.
4. Distracting the child was more likely to occur if the parents were internal.
5. Externally oriented mothers are more likely to shout at or smack their child when he or she is having a tantrum. The father's orientation makes little difference to whether the mother shouts at or smacks the child who is having a tantrum if she is external, but it is associated with a difference in such behavior if she is internal.
6. Finally we examine the frequency of having tantrums at 57 months. The children of external mothers had more frequent temper tantrums than the children of internal mothers; the locus of control of the father appeared to make little or no contribution to the occurrence of tantrums.

### General observations

Prenatal parent externality is associated with the consistent parental report of more negative child outcomes than is prenatal parent internality. However the association may be due to external parents having a more negative self-reporting bias than their internal counterparts and it is the bias rather than actual behavioral differences that is responsible for the greater report of problematic child outcomes. One way to evaluate this possibility would be to gather observations of children's behavior from adults other than parents. Teachers are likely candidates. Teacher ratings of children's personality characteristics and psychological functioning are free from the potential negative self-reporting bias of external parents and could provide unbiased evidence of the differential impact of parental locus of control on children's personal and social behavior.

It is apparent that, at the preschool ages focused on in this study, children with externally controlled parents had more difficulty establishing satisfactory eating and sleeping routines compared to their peers with internally controlled parents. Even before the birth of the child, externally controlled parents were less likely to take advantage of programs that could have better prepared them to deal with a new infant, and when an infant arrived they consistently showed that they were less able to organize and direct their children in their everyday activities like sleeping, eating, or loss of temper. This consistent pattern of inconsistent and relatively unstructured parenting surrounding bedtime and meal time may produce children who are less prepared for leaving home to attend full time school. They, both boys and girls with external parents, would be expected to have more problems in school paying attention, following directions, and interacting successfully with others. Future research on children's behavior outside the home when they are older is needed to evaluate this expectation.

How can children of external parents be helped to deal with the lack of structure and consistency they experience? One possibility is by supporting and educating their parents. It is likely that the organizational miscues external parents make may originate from a lack of knowledge of what to expect and what to do when the child arrives in their lives. External parents should be encouraged in every way possible to prepare themselves better for the arrival of their children. External parents may not perceive the connection between what they might learn from prenatal classes and other preparatory experiences that would benefit them in dealing with their child. Because past research shows that externals learn best in structured situations and are especially responsive to primary reinforcement, preparatory programs should be used that take these factors into account.

Besides attempts to improve the child caring knowledge and skill of externally controlled parents, interventions could also focus on directly changing their locus of control orientation toward internality. Some past programs have been marginally successful at changing locus of control in large communities of adults (e.g., Knapp and McClure, 1978), others have been more effective by focusing on changing the language (Roueche and Mink, 1976) or the cognitive thinking (Wolinsky et al., 2010) of individuals. At the same time, it might be useful to apply educational programs that help children become appropriately internal (e.g., Nowicki et al., 2004). Past research has shown that children's internality provides protection from their feelings of helplessness and depression later in childhood. For example Culpin et al. (2015) found that children from impoverished backgrounds who were at risk for developing depression were able to avoid this outcome if they became internally controlled during their adolescence. Programs instituted to change younger children toward internality may make them more resilient in dealing with negative forces around them.

### Strengths and limitations

The strengths of this project lie in (a) the use of a prospective design; (b) the use of a large and representative population of participants; and (c) the inclusion of both mother's and father's locus of control. This study is the first to examine the associations of parent locus of control and child outcomes over such an extended period of time and provide substantial evidence of the developmental effects of parent internality and externality on children.

Having said that there are a number of limitations. One is that the analyses were restricted to the 80% of the eligible pregnant population that took part in the study. Those who did not take part were biased in that they were likely to be teenagers and/or of low educational achievements (features that have been found associated with externality). However, Fraser et al. (2013) noted that the demographic differences were relatively small.

Another limitation is that the present study was prospective in design, but not a completely longitudinal investigation, since we did not measure parental LOC again during the child's preschool years. It is also possible that as well as parents affecting the children's outcomes, children may have impacted on parents as well. One way to evaluate this possibility also points out another limitation of the present study; there was no direct observation of what went on between parents and children for any of the outcomes. Some studies have directly observed how internal and external parents behaved when interacting with their children (Carton, 1996). The same design could be used to examine the behavior of internal and external parents with their children, especially if both parents were involved in the study.

## Conclusion

Parental locus of control has much to do with how parents interact with their children. It is clear from the present study that parental locus of control is associated with a number of important parent child interactions surrounding social contact, sleeping, eating, and behavioral regulation. The greater the internality of the parents the better the child outcomes, the greater the externality the worse the child outcomes are in all of these crucial areas except for picky eating in the pre-school child. We suggest that initiatives be made to help parents develop a more internal locus of control with the goal of improving their ability to parent effectively.

## Ethical approval

Ethical approval for the study was obtained from the ALSPAC Ethics and Law Committee and the Local Research Ethics Committees.

## Author contributions

SN had the idea, JG planned and carried out the analyses with GE and SG; SN and JG wrote the first draft; all were involved in editing, checking and rewriting the paper.

## Funding

The UK Medical Research Council and the Wellcome Trust (Grant ref: 102215/2/13/2) and the University of Bristol currently provide core support for ALSPAC. This publication is the work of the authors and they will serve as guarantors for the contents of this paper. This research was specifically funded by a grant from the John Templeton Foundation (Grant Ref: 58223).

### Conflict of interest statement

The authors declare that the research was conducted in the absence of any commercial or financial relationships that could be construed as a potential conflict of interest.
