# Supplementary material for: The Impact of Prenatal Parental Locus of Control on Children's Psychological Outcomes in Infancy and Early Childhood: A Prospective 5 Year Study
Source: Front Psychol. 2017 Apr 12;8:546. doi: 10.3389/fpsyg.2017.00546 (PMC5388778; doi:10.3389/fpsyg.2017.00546)
Supplement: Supplementary file 1 [file Table1.DOCX]

**SUPPLEMENTARY TABLES**

Table S1. Attitudes and behaviors of parents according to the LOC orientation of each parent

| Behavior | N | M.Ext | M.Int | OR[95%CI] | N | F.Ext | F.Int | OR [95% CI] |
| --- | --- | --- | --- | --- | --- | --- | --- | --- |
|  |  | % | % |  |  | % | % |  |
| M uncertain of  date of LMP | 11979 | 19.1 | 13.2 | 1.56[1.41,1.72] | 8332 | 17.4 | 11.8 | 1.56[1.39,1.79] |
|  |  |  |  |  |  |  |  |  |
| M did not attend  AN/parenting classes | 11312 | 44.8 | 35.4 | 1.47[1.37,1.59] | 7999 | 40.2 | 34.0 | 1.30[1.19,1.43] |
|  |  |  |  |  |  |  |  |  |
| F did not attend  classes | 11233 | 79.2 | 68.1 | 1.79[1.64,1.96] | 7966 | 74.3 | 63.6 | 1.64[1.49,1.82] |
|  |  |  |  |  |  |  |  |  |
| Father was not  present at delivery | 11349 | 15.8 | 10.6 | 1.59[1.43,1.79] | 8021 | 13.5 | 10.2 | 1.37[1.19,1.56] |
|  |  |  |  |  |  |  |  |  |
| Child not breast fed | 11621 | 31.5 | 15.0 | 2.61[2.38,2.85] | 8200 | 28.2 | 14.5 | 2.31[2.07,2.58] |
|  |  |  |  |  |  |  |  |  |
| Child not fully  immunized by 6m | 11038 | 7.0 | 4.5 | 1.96[1.75,2.17] | 7861 | 13.8 | 8.7 | 1.67[1.45,1.92] |

Table S2. Child **does not have regular sleeping routine**: Percentage of each group of mothers who have not achieved a successful sleeping routine with their baby according to the locus of control orientation of each parent

| Age | N | M.Ext | M.Int | OR[95%CI] | N | F.Ext | F.Int | OR[95%CI] |
| --- | --- | --- | --- | --- | --- | --- | --- | --- |
|  |  | % | % |  |  | % | % |  |
|  |  |  |  |  |  |  |  |  |
| 18m | 10612 | 16.4 | 11.7 | 1.48 [1.33, 1.66]^d^ | 7613 | 15.7 | 10.4 | 1.61 [1.40, 1.84]^d^ |
| 30m | 9887 | 14.7 | 8.4 | 1.87 [1.65,2.12]^d^ | 7154 | 13.2 | 8.5 | 1.64 [1.41,1.84]^d^ |
| 42m | 9620 | 10.8 | 5.6 | 2.02 [1.73. 2.35]^d^ | 6999 | 9.1 | 5.7 | 1.67 [1.39,2.00]^d^ |
| 57m | 9112 | 8.2 | 3.8 | 2.25 [1.87, 2.70]^d^ | 6669 | 6.4 | 4.1 | 1.59 [1.28, 1.98]^d^ |

^d^P < 0.0001

Table S3. Child regularly **refuses to go to bed**: Percentage of each group of mothers who have not achieved a successful sleeping strategy with their baby according to the locus of control orientation of each parent

| Age | N | M.Ext | M.Int | OR[95%CI] | N | F.Ext | F.Int | OR[95%CI] |
| --- | --- | --- | --- | --- | --- | --- | --- | --- |
|  |  | % | % |  |  | % | % |  |
| 18m | 10589 | 29.7 | 22.7 | 1.43 [1.31, 1.56]^d^ | 7607 | 27.3 | 22.2 | 1.32 [1.19, 1.46]^d^ |
| 30m | 9861 | 53.2 | 40.7 | 1.66 [1.53, 1.79]^d^ | 7146 | 50.0 | 39.5 | 1.53 [1.40, 1.69]^d^ |
| 42m | 9690 | 51.0 | 37.3 | 1.75 1.61, 1.90]^d^ | 7052 | 46.4 | 35.5 | 1.57 [1.43, 1.73]^d^ |
| 57m | 9108 | 47.8 | 33.4 | 1.83 [1.68, 2.00]^d^ | 6665 | 43.0 | 32.7 | 1.55 1.41, 1.72]^d^ |

^d^P < 0.0001

Table S4. Child regularly **gets up after only a few hours’ sleep**. Percentage of each group of mothers who have not achieved a successful sleep strategy with their baby according to the locus of control orientation of each parent

| Age | N | M.Ext | M.Int | OR[95%CI] | N | F.Ext | F.Int | OR[95%CI] |
| --- | --- | --- | --- | --- | --- | --- | --- | --- |
|  |  | % | % |  |  | % | % |  |
| 18m | 10,589 | 25.2 | 17.9 | 1.54 [1.40, 1.69]^d^ | 7607 | 23.1 | 17.1 | 1.46 [1.30, 1.63]^d^ |
| 30m | 9811 | 26.9 | 19.0 | 1.57 [1.43,1.73]^d^ | 7107 | 24.5 | 18.8 | 1.40 [1.25,1.57]^d^ |
| 42m | 9690 | 21.1 | 14.1 | 1.63 [1.46. 1.81]^d^ | 7052 | 19.1 | 13.8 | 1.47 [1.30,1.67]^d^ |
| 57m | 9062 | 14.9 | 10.1 | 1.56 [1.37, 1.77]^d^ | 6637 | 13.9 | 9.4 | 1.54 [1.33, 1.80]^d^ |

^d^P < 0.0001

Table S5. Mother finds it **difficult to establish an eating routine**. Percentage of each group of mothers who have not achieved a successful eating routine with their baby according to the locus of control orientation of each parent

| Age | N | M.Ext | M.Int | OR[95%CI] | N | F.Ext | F.Int | OR[95%CI] |
| --- | --- | --- | --- | --- | --- | --- | --- | --- |
|  |  | % | % |  |  | % | % |  |
| 24m | 10,059 | 22.9 | 21.0 | 1.12 [1.02, 1.23]^a^ | 7271 | 21.9 | 21.3 | 1.03 [0.92, 1.16]^-^ |
| 38m | 9775 | 28.1 | 24.4 | 1.21 [1.11, 1.33]^d^ | 7099 | 26.3 | 24.2 | 1.11 [1.00,1.24]^a^ |
| 54m | 9173 | 23.5 | 18.5 | 1.35 [1.22, 1.50]^d^ | 6734 | 21.0 | 18.8 | 1.15 [1.02, 1.30]^a^ |

^a^P < 0.05; ^d^P < 0.0001

Table S6. Child has **overeaten** in past year. Percentage of mothers who reported their child as having over-eaten according to the locus of control orientation of each parent

| Age | N | M.Ext | M.Int | OR[95%CI] | N | F.Ext | F.Int | OR[95%CI] |
| --- | --- | --- | --- | --- | --- | --- | --- | --- |
|  |  | % | % |  |  | % | % |  |
| 15m | 10,615 | 22.1 | 18.6 | 1.23 [1.12, 1.36]^d^ | 7617 | 21.8 | 17.9 | 1.27 [1.14, 1.43]^d^ |
| 24m | 10,059 | 18.1 | 15.3 | 1.23 [1.10, 1.36]^c^ | 7271 | 17.8 | 14.7 | 1.26 [1.11, 1.42]^c^ |
| 38m | 9775 | 16.1 | 13.0 | 1.28 [1.14, 1.44]^d^ | 7099 | 15.5 | 12.7 | 1.27 [1.11,1.45]^c^ |
| 54m | 9183 | 19.5 | 15.0 | 1.38 [1.23, 1.54]^d^ | 6745 | 18.9 | 15.1 | 1.31 [1.15,1.49]^d^ |

^c^P < 0.001; ^d^P < 0.0001

Table S7. Child has been **choosy with food** in past year. Percentage of mothers who reported their child as having been choosy with food according to the locus of control orientation of each parent

| Age | N | M.Ext | M.Int | OR[95%CI] | N | F.Ext | F.Int | OR[95%CI] |
| --- | --- | --- | --- | --- | --- | --- | --- | --- |
|  |  | % | % |  |  | % | % |  |
| 15m | 10,615 | 51.9 | 57.5 | 0.80 [0.74, 0.86]^d^ | 7617 | 53.7 | 57.0 | 0.88 [0.80, 0.96]^b^ |
| 24m | 10,059 | 65.7 | 70.2 | 0.81 [0.75, 0.88]^d^ | 7271 | 66.5 | 70.0 | 0.85 [0.77, 0.94]^b^ |
| 38m | 9775 | 70.7 | 74.5 | 0.83 [0.75,0.90]^d^ | 7099 | 72.4 | 73.3 | 0.95 [0.86, 1.06]^-^ |
| 54m | 9224 | 77.9 | 80.5 | 0.85 [0.76, 0.94]^b^ | 6770 | 78.5 | 79.9 | 0.93 [0.82, 1.03]^-^ |

^b^P , 0.01; ^d^P < 0.0001

S8. Child’s ability to choose what to eat for the **main meal** at 57m

| CHOICE | M.Ext | M.Int | OR [95%CI] | F.Ext | F.Int | OR [95%CI] |
| --- | --- | --- | --- | --- | --- | --- |
|  | % | % |  | % | % |  |
| From any available foods | 18.9 | 10.2 | 2.04 [1.79, 2.31] | 15.6 | 11.1 | 2.04 [1.79,2.31] |
|  | 46.1 | 50.8 | 1.00 Ref | 49.6 | 48.7 | 1 Ref |
| Adult decides | 35.0 | 39.0 | 0.99 [0.90, 1.08] | 34.8 | 40.3 | 0.99 [0.90,1.08] |
|  |  |  | P < 0.0001  (n=8964) |  |  |  |

Table S9. Child’s ability to choose what **snacks** to eat

| CHOICE | M.Ext | M.Int | OR[95%CI] | F.Ext | F.Int | OR[95%CI] |
| --- | --- | --- | --- | --- | --- | --- |
|  | % | % |  | % | % |  |
| From any available snacks | 49.2 | 34.9 | 1.90 [1.73, 2.07] | 45.7 | 33.7 | 1.67 [1.50, 1.85] |
| From a few alternatives | 42.4 | 56.9 | 1.00 Ref | 46.5 | 57.1 | 1.00 Ref |
| Adult decides | 8.4 | 8.3 | 1.36 [1.16, 1.60] | 7.8 | 9.1 | 1.05 [0.87, 1.26] |
|  |  |  | P < 0.0001  (n = 8818) |  |  | P < 0.0001  (n = 6450) |

Table S10. Frequency with which mother **ignores temper tantrum**

| FREQUENCY | M.Ext. | M.Int. | OR[95%CI] | F.Ext | F.Int. | OR[95%CI] |
| --- | --- | --- | --- | --- | --- | --- |
|  | % | % |  | % | % |  |
| ***At 18m*** |  |  |  |  |  |  |
| Often | 50.8 | 46.0 | 1.18 [1.09, 1.28] | 48.9 | 47.0 | 1.05 [0.95, 1.16] |
| Sometimes | 42.7 | 45.5 | 1.00 Ref | 44.5 | 45.0 | 1.00 Ref |
| Never | 6.5 | 8.5 | 0.81 [0.68, 0.95] | 6.5 | 8.1 | 0.82 [0.67, 1.00] |
|  |  |  | P < 0.0001 (n =9308) |  |  | P = 0.023 (n = 6654) |
| ***At 30m*** |  |  |  |  |  |  |
| Often | 44.9 | 40.0 | 1.19 [1.09, 1.30] | 43.9 | 39.3 | 1.18 [1.07, 1.31] |
| Sometimes | 50.9 | 54.1 | 1.00 Ref | 51.6 | 54.8 | 1.00 Ref |
| Never | 4.2 | 6.0 | 0.75 [0.62, 0.92] | 4.5 | 5.9 | 0.82 [0.65, 1.04] |
|  |  |  | P < 0.0001 (n = 8616 |  |  | P< 0.0001 (n = 6225) |
| ***At 42m*** |  |  |  |  |  |  |
| Often | 38.6 | 32.1 | 1.33 [1.21, 1.46] | 36.3 | 32.0 | 1.20 [1.07, 1.34] |
| Sometimes | 52.1 | 57.6 | 1.00 Ref | 54.7 | 57.9 | 1.00 Ref |
| Never | 9.4 | 10.3 | 1.01 [0.87, 1.18] | 9.0 | 10.1 | 0.95 [0.79, 1.13] |
|  |  |  | P < 0.0001 (n = 7953) |  |  | P= 0.001 (n = 5782) |

Table S11. Frequency with which she **cuddles** the child during temper tantrum

| FREQUENCY | M.Ext | M.Int | OR[95%CI] | F.Ext | F.Int | OR[95%CI] |
| --- | --- | --- | --- | --- | --- | --- |
|  | % | % |  | % | % |  |
| ***At 18m*** |  |  |  |  |  |  |
| Often | 16.5 | 18.2 | 0.96 [0.86, 1.08] | 15.8 | 18.9 | 0.86 [0.75, 0.98] |
| Sometimes | 55.2 | 58.7 | 1.00 Ref | 56.3 | 58.0 | 1.00 Ref |
| Never | 28.3 | 23.0 | 1.31 [1.19, 1.44] | 27.9 | 23.1 | 1.24 [1.11, 1.39] |
|  |  |  | P <0.0001  (n = 9308) |  |  | P < 0.0001  (n = 6654) |
| ***At 30m*** |  |  |  |  |  |  |
| Often | 17.0 | 17.6 | 1.03 [0.91, 1.15] | 16.4 | 17.8 | 0.95 [0.83, 1.09] |
| Sometimes | 59.9 | 63.4 | 1.00 Ref | 61.0 | 62.9 | 1.00 Ref |
| Never | 23.1 | 19.0 | 1.29 [1.15, 1.44] | 22.6 | 19.2 | 1.21 [1.07, 1.38] |
|  |  |  | P < 0.0001  (n = 8408) |  |  | P < 0.0001  (n = 6083) |
| ***At 42m*** |  |  |  |  |  |  |
| Often | 12.4 | 13.4 | 1.01 [0.88, 1.15] | 12.2 | 13.1 | 1.00 [0.85, 1.18] |
| Sometimes | 54.5 | 59.4 | 1.00 Ref | 55.5 | 60.0 | 1.00 Ref |
| Never | 33.1 | 27.2 | 1.29 [1.15, 1.44] | 32.3 | 26.9 | 1.30 [1.15, 1.46] |
|  |  |  | P < 0.0001  (n = 7953 |  |  | P < 0.001  (n = 6654) |

Table S12. Frequency with which **parents reason** with the child who is having a temper tantrum

| FREQUENCY | M.Ext | M.Int | OR[95%CI] | F.Ext | F.Int | OR[95%CI] |
| --- | --- | --- | --- | --- | --- | --- |
|  | % | % |  | % | % |  |
| ***At 18m*** |  |  |  |  |  |  |
| Often | 21.5 | 21.9 | 1.00 [0.90, 1.11] | 22.1 | 221.2 | 1.02 [0.90, 1.15] |
| Sometimes | 53.1 | 54.1 | 1.00 Ref | 55.1 | 53.8 | 1.00 Ref |
| Never | 25.4 | 24.0 | 1.08 [0.98, 1.19] | 22.8 | 25.0 | 0.89 [0.79, 1.01] |
|  |  |  | P = 0.214  (n = 9308) |  |  | P = 0.068  (n = 6654) |
| ***At 30m*** |  |  |  |  |  |  |
| Often | 32.8 | 33.9 | 0.96 [0.87, 1.05] | 33.9 | 33.2 | 1.04 [0.93, 1.16] |
| Sometimes | 59.4 | 58.8 | 1.00 Ref | 58.5 | 59.5 | 1.00 Ref |
| Never | 7.8 | 7.2 | 1.07 [0.91, 1.27] | 7.6 | 7.3 | 1.04 [0.86, 1.27] |
|  |  |  | P = 0.181  (n = 8502) |  |  | P = 0.706  (n = 6143) |
| ***At 42m*** |  |  |  |  |  |  |
| Often | 34.6 | 36.3 | 0.95 [0.86, 1.04] | 37.0 | 35.3 | 1.10 [0.99, 1.23] |
| Sometimes | 55.3 | 55.1 | 1.00 Ref | 53.4 | 56.2 | 1.00 Ref |
| Never | 10.1 | 8.5 | 1.18 [1.01, 1.38] | 9.7 | 8.4 | 1.21 [1.01, 1.46] |
|  |  |  | P = 0.016  (n=7953) |  |  | P = 0.814  (n = 5782) |

Table S13. Frequency with which mother tries to **distract** the child during temper tantrum

| FREQUENCY | M.Ext | M.Int | OR[95%CI] | F.Ext | F.Int | OR[95%CI] |
| --- | --- | --- | --- | --- | --- | --- |
|  | % | % |  | % | % |  |
| ***At 18m*** |  |  |  |  |  |  |
| Often | 41.0 | 52.5 | 0.69 [0.63, 0.75] | 43.9 | 51.6 | 0.79 [0.71, 0.91 |
| Sometimes | 46.1 | 40.9 | 1.00 Ref | 44.8 | 41.6 | 1.00 Ref |
| Never | 12.9 | 6.6 | 1.76 [1.51, 2.03] | 11.3 | 6.8 | 1.54 [1.29, 1.84] |
|  |  |  | P < 0.0001  (n = 9308) |  |  | P < 0.0001  (n = 6654) |
| ***At 30m*** |  |  |  |  |  |  |
| Often | 35.5 | 41.8 | 0.81 [0.74, 0.89] | 37.0 | 42.0 | 0.86 [0.77, 0.96] |
| Sometimes | 54.8 | 52.7 | 1.00 Ref | 53.9 | 52.7 | 1.00 Ref |
| Never | 9.8 | 5.5 | 1.72 [1.45, 2.04] | 9.1 | 5.3 | 1.67 [1.36, 2.04] |
|  |  |  | P < 0.0001  (n = 8552) |  |  | P <0.0001  (n = 6179) |
| ***At 42m*** |  |  |  |  |  |  |
| Often | 25.6 | 31.2 | 0.82 [0.74, 0.91] | 27.5 | 29.8 | 0.95 [0.85, 1.07] |
| Sometimes | 56.5 | 56.5 | 1.00 Ref | 55.8 | 57.7 | 1.00 Ref |
| Never | 17.9 | 12.4 | 1.45 [1.27, 1.65] | 16.7 | 12.5 | 1.39 [1.19, 1.62] |
|  |  |  | P <0.0001  (n=7953) |  |  | P < 0.0001  (n = 5782) |

Table S14. Frequency mother **smacks** the child when having a tantrum (18m)

| FREQUENCY | M.Ext | M.Int | OR[95%CI] | F.Ext | F.Int | OR[95%CI] |
| --- | --- | --- | --- | --- | --- | --- |
|  | % | % |  | % | % |  |
| Often | 1.0 | 0.4 | 1.76 [1.05, 2.95] | 1.0 | 0.4 | 2.10 [1.13, 3.90] |
| Sometimes | 25.9 | 20.5 | 1.00 Ref | 24.2 | 20.8 | 1.00 Ref |
| Never | 73.1 | 79.1 | 0.73 [0.66, 0.81] | 74.8 | 78.8 | 0.82 [0.73, 0.89] |
|  |  |  | P< 0.0001  (n =9308) |  |  | P < 0.0001  (n = 6654) |

Table S15. Frequency mother **shouts** at the child when having a tantrum (18m)

| FREQUENCY | M.Ext | M.Int | OR[95%CI] | F.Ext | F.Int | OR[95%CI] |
| --- | --- | --- | --- | --- | --- | --- |
|  | % | % |  | % | % |  |
| Often | 4.2 | 2.4 | 1.56 [1.23, 1.97] | 4.0 | 2.5 | 1.50 [1.14, 1.99] |
| Sometimes | 59.0 | 52.6 | 1.00 Ref | 56.6 | 53.7 | 1.00 Ref |
| Never | 36.8 | 45.0 | 0.73 [0.67, 0.79] | 39.4 | 43.8 | 0.85 [0.77, 0.94] |
|  |  |  | P< 0.0001  (n =9308) |  |  | P < 0.0001  (n = 6654) |
